# Novel Pharmacological Options in the Treatment of Cholangiocarcinoma: Mechanisms of Resistance

**DOI:** 10.3390/cancers13102358

**Published:** 2021-05-13

**Authors:** Jose J. G. Marin, Paula Sanchon-Sanchez, Candela Cives-Losada, Sofía del Carmen, Jesús M. González-Santiago, Maria J. Monte, Rocio I. R. Macias

**Affiliations:** 1 Experimental Hepatology and Drug Targeting (HEVEPHARM) Group, Biomedical Research Institute (IBSAL), University of Salamanca, 37007 Salamanca, Spain; pausanchons@usal.es (P.S.-S.); candelacives@usal.es (C.C.-L.); mjmonte@usal.es (M.J.M.); 2 Centro de Investigación Biomédica en Red para el Estudio de Enfermedades Hepáticas y Digestivas (CIBERehd), Carlos III National Institute of Health, 28029 Madrid, Spain; 3 Department of Pathology, Biomedical Research Institute (IBSAL), University Hospital of Salamanca, 37007 Salamanca, Spain; sofia_delcarmen@hotmail.com or; 4 Department of Gastroenterology and Hepatology, Biomedical Research Institute (IBSAL), University Hospital of Salamanca, 37007 Salamanca, Spain; jmgsantiago@saludcastillayleon.es

**Keywords:** biliary cancer, immunotherapy, pharmacoresistance, targeted therapy

## Abstract

**Simple Summary:**

Cholangiocarcinoma, a tumor derived from epithelial cells of the biliary tree, is characterized by a dismal prognosis. Its late diagnosis, which makes surgical resection not an option for most patients, and its marked refractoriness to standard chemotherapy, justify its high position in the rank of the most lethal cancers. Identifying specific druggable genetic alterations constitutes a promising alternative for the use of personalized targeted anticancer agents, and immunotherapy, or drugs able to interact with proteins involved in the crosstalk between cancer and immune cells, could also be an option in the future. However, it has also been observed that some patients fail to respond to these new therapies or after an initial response, the disease progresses. Therefore, understanding the mechanisms of pharmacoresistance is of utmost importance to design more effective treatments.

**Abstract:**

Despite the crucial advances in understanding the biology of cholangiocarcinoma (CCA) achieved during the last decade, very little of this knowledge has been translated into clinical practice. Thus, CCA prognosis is among the most dismal of solid tumors. The reason is the frequent late diagnosis of this form of cancer, which makes surgical removal of the tumor impossible, together with the poor response to standard chemotherapy and targeted therapy with inhibitors of tyrosine kinase receptors. The discovery of genetic alterations with an impact on the malignant characteristics of CCA, such as proliferation, invasiveness, and the ability to generate metastases, has led to envisage to treat these patients with selective inhibitors of mutated proteins. Moreover, the hope of developing new tools to improve the dismal outcome of patients with advanced CCA also includes the use of small molecules and antibodies able to interact with proteins involved in the crosstalk between cancer and immune cells with the aim of enhancing the immune system’s attack against the tumor. The lack of effect of these new therapies in some patients with CCA is associated with the ability of tumor cells to continuously adapt to the pharmacological pressure by developing different mechanisms of resistance. However, the available information about these mechanisms for the new drugs and how they evolve is still limited.

## 1. Introduction

Cholangiocarcinoma (CCA) is the second-most common cancer affecting the hepatobiliary system. The term refers to a heterogeneous group of highly malignant tumors generated from epithelial cells of the biliary tree. Based on their anatomical site of origin, CCAs can be classified into three subtypes: intrahepatic (iCCA), perihilar (pCCA), and distal (dCCA), which are now considered different oncological entities [1]. Their incidence and mortality are increasing worldwide. CCAs are characterized by a silent evolution that determines that only 30% of patients present localized disease at diagnosis and are therefore candidates for surgical resection; nevertheless, they frequently (42–70% of cases) relapse after surgery, even when patients have received adjuvant chemotherapy [2]. Liver transplant has shown an acceptably good overall survival (OS) in cirrhotic iCCA patients diagnosed at a very early stage [3]. Still, to date, the number of cases that could benefit from this treatment is low, as it is also the number of patients that are candidates for locoregional therapies.

**Figure 1:**
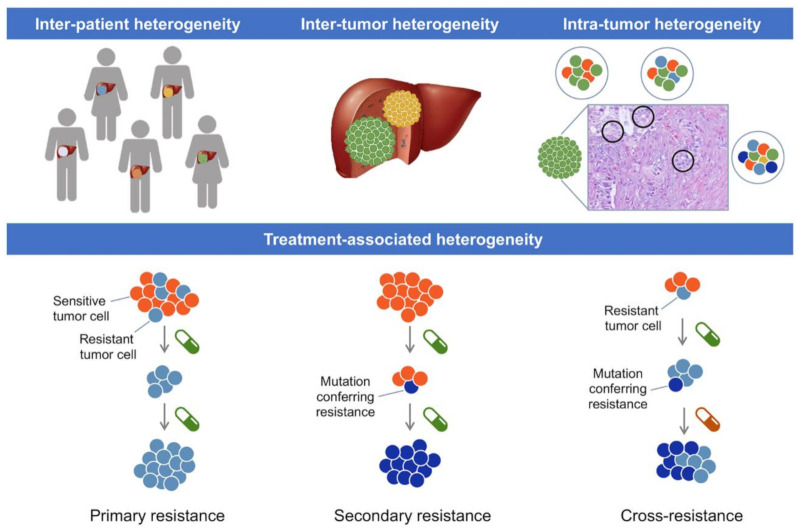
Schematic representation of inter-patient, inter-tumor and intra-tumor heterogeneity in CCA, as well as tumor heterogeneity in response to pharmacological treatment.

The prognosis of unresectable CCA patients is dismal because these tumors are highly resistant to the classic systemic pharmacological regimes based on gemcitabine (2′,2′-difluorodeoxycytidine), cisplatin, and 5-fluorouracil (5-FU), resulting in less than two years of OS. There is substantial genomic heterogeneity in tumors from different patients [4], which explains the different responses to chemotherapy. Moreover, there is also genomic diversity among cells within a tumor, which accounts for two frequent features; (i) an initial partial response followed by the development of drug resistance due to the growth of tumor cells with characteristics that permit them to avoid the toxic effect of the drugs, and (ii) the appearance of de novo mutations over time in some drug-sensitive tumor cell subpopulations that help them survive and also contribute to drug resistance (Figure 1).

The mechanisms of pharmacoresistance (MPRs) can be present before starting the treatment (primary pharmacoresistance) or can be developed during exposure to drugs (acquired pharmacoresistance). MPRs have been divided into seven groups according to changes in proteins responsible for (i) reduced amount of the intracellular active drug due to decreased uptake or enhanced efflux (MPR-1); (ii) altered drug/prodrug metabolism (MPR-2); (iii) changes in the molecular targets (MPR-3); (iv) enhanced repair of drug-induced DNA damage (MPR-4); (v) altered apoptosis/survival balance (MPR-5); (vi) changes in the tumor microenvironment (MPR-6), and (vii) phenotypic transition with the development of mesenchymal or stemness characteristics (MPR-7). The existence of MPRs affecting the usefulness of classical chemotherapeutical agents commonly used in CCA treatment has been previously revised [5,6]. The efficacy of these MPRs accounts for CCA refractoriness to these drugs and justifies the need to identify more successful drugs.

The vast genomic heterogeneity of biliary tract cancers (BTCs), with different mutations in each BTC subtype [7], is a challenge that makes it difficult to find single treatments useful for all the patients. However, it also provides opportunities to design tailored therapies that usually target specific alterations that drive proliferation and survival in tumor cells. Among these new therapies, kinase inhibitors acting on several enzymes and receptors, hybrid molecules directed to targets involved in various pathways, and combinations of drugs with different mechanisms of action are being investigated in clinical trials [8].

In this review, the MPRs to the novel drugs that have shown promising efficacy for patients with CCA have been revised, which mainly include targeted therapies and immunotherapies. They have been compared with those described for classical chemotherapy to elucidate whether tumor cells use similar mechanisms to evade the toxic effects of these novel drugs or new MPRs are involved.

## 2. CCA Resistance to Classical Chemotherapy

The combination of gemcitabine and platinum-derived drugs is still recognized as the first-line treatment option for patients with advanced or metastatic CCA. Traditionally, gemcitabine monotherapy was used to treat these patients until 2010, where two separated clinical trials revealed that, compared to gemcitabine alone, cisplatin plus gemcitabine was associated with a significant survival advantage, without the addition of substantial toxicity [9,10]. Despite being the current standard of pharmacological treatment, the efficacy of first-line cisplatin–gemcitabine chemotherapy in patients with advanced iCCA is limited, with median progression-free survival (PFS) and OS of 8.4 and 15.4 months, respectively [11]. Moreover, this regimen is associated with side effects (mainly nausea, vomiting, and anorexia) and the development of pharmacoresistance. When cisplatin is contraindicated, for example, in patients with renal insufficiency, it is replaced by oxaliplatin. Chemotherapy with gemcitabine and oxaliplatin (GEMOX regimen) is better tolerated and demonstrated modest antitumor activity in patients with advanced BTCs [12].

It is controversial whether second-line chemotherapy can provide clinical benefit in advanced biliary cancer [13,14], although several studies have shown some degree of efficacy in selected CCA patients. Fluoropyrimidines such as 5-FU, capecitabine (an oral prodrug of 5-FU) and S-1 (an oral combination of tegafur, gimeracil, and oteracil) are commonly used in clinical practice when gemcitabine-based treatments fail.

Regarding adjuvant chemotherapy, capecitabine can improve OS in patients with resected BTC when used after surgery and hence is considered the standard of care. Thus, international guidelines recommend the administration of adjuvant capecitabine for a period of six months following potentially curative resection of CCA [8,15].

Several MPRs can act synergistically to induce the failure of classical chemotherapy used in the treatment of CCA (Table 1). This has been extensively studied in the case of gemcitabine. Because this anticancer agent is a nucleoside analog of deoxycytidine, human equilibrative nucleoside transporter 1 (ENT-1) is the major carrier involved in the uptake of this drug by cancer cells. ENT-1 expression and subcellular localization may predict the degree of response to this drug in CCA and advanced BTC, as treated patients harboring ENT-1 on the tumor cell membrane had longer disease-free survival compared with patients lacking ENT-1 or expressing this protein, but failing in its targeting to the plasma membrane of tumor cells [16,17].

On the other hand, gemcitabine-resistant CCA cell lines KKUM139/GEM and KKUM214/GEM obtained by stepwise exposure to increasing gemcitabine concentrations retained the resistant phenotype in a drug-free medium at least for two months. This included upregulation of multidrug resistance-associated protein 1 (MRP1), leading to enhanced drug efflux [18]. MRP1 expression (but not that of other ABC pumps) was found significantly higher in iCCA tissues than in paired non-tumor tissues [31], suggesting that this efflux carrier may be involved in gemcitabine resistance. However, this point has not been demonstrated for CCA in the clinical setting. Once transported into the cell, gemcitabine must be phosphorylated by deoxycytidine kinase (DCK) to its active form, and both gemcitabine diphosphate and gemcitabine triphosphate can impair DNA synthesis, causing chain termination and leading to apoptosis activation [32]. DCK enzymatic activity, and therefore intracellular drug activation, is decreased in gemcitabine-resistant CCA cell lines [18]. Mechanisms involved in DNA repair can also account for resistance to gemcitabine. Thus, an increased expression of the p53-inducible ribonucleotide reductase (P53R2), required for efficient DNA repair, may predict gemcitabine resistance in CCA [19]. Karyopherin-α2 (KPNA2) mediates nuclear translocation of an essential component of the DNA repair machinery, the MRE11-RAD50-NBS1 (MRN) complex. In CCA patients who received gemcitabine after surgery, KPNA2 overexpression has been suggested to be a prognostic indicator of poor OS [20]. The upregulation of the anti-apoptotic protein Bcl-2 and downregulation of the pro-apoptotic one Bax, altogether leading to evasion from apoptosis, in association with higher activity of matrix metalloproteinase-9 (MMP-9) and urokinase plasminogen activator (uPA), resulting in higher migration and invasion capacities, were also observed in gemcitabine-resistant CCA cell lines [18].

Tumor microenvironment conditions, such as hypoxia, nutrient starvation, and acidic extracellular pH, play critical roles in pharmacoresistance. CCA cells can adapt to an acidic environment by upregulating octamer-binding transcription factor 4 (Oct4), and this upregulation has been associated with gemcitabine resistance in CCA cell lines [21]. High mobility group A1 (HMGA1) proteins promote proliferation and epithelial-mesenchymal transition (EMT) of tumor cells. HMGA1 expression is increased in a substantial number of CCA specimens, and it has been proposed both to enhance CCA tumorigenicity and to confer resistance to gemcitabine [22]. Finally, crosstalk between interleukin-6 (IL-6) and transforming growth factor-beta 1 (TGF-β1) signaling pathways has also been shown to play a role in EMT and gemcitabine resistance in BTC patients [23].

Aiming to overcome the critical MPRs accounting for gemcitabine resistance, NUC-1031, a phosphoramidate derivative of gemcitabine, has been specifically designed to reach higher concentrations of the active drug inside tumor cells. The phosphoramidate moiety enables NUC-1031 to enter the cell regardless of ENT-1 expression. Once inside, the protective group is cleaved off, resulting in the release of an activated (monophosphorylated) form of gemcitabine, which prevents the need for DCK activity. Besides, the active drug is less sensitive to metabolic inactivation. A Phase Ib study comparing the efficacy of the combination of NUC-1031 and cisplatin with that of gemcitabine and cisplatin as first-line treatment in patients with advanced BTC has shown promising results when using this new drug [33].

The activity of enzymes related to 5-FU metabolism may predict the sensitivity to fluoropyrimidine-based drugs. Thus, the mRNA expression of orotate phosphoribosyl transferase (OPRT), a key enzyme in the intracellular activation of 5-FU, is significantly lower in CCA tissues non-responding to this drug than in the responder group [24]. Increased expression of thymidylate synthase (TS), which is involved in DNA synthesis and inhibited by 5-FU metabolites, impairs the sensitivity of BTC cells to 5-FU [25]. Modulation of apoptosis can also have a role in resistance to 5-FU in CCA cells. NK4, an antagonist for the hepatocyte growth factor (HGF) and the MET receptor, is involved in the 5-FU-induction of apoptosis through the intrinsic mitochondrial pathway. Downregulation of NK4 in response to 5-FU may represent an intrinsic mechanism of resistance to this anticancer drug [26].

Among MPRs affecting the response of CCA to platinum derivatives, reduced levels of organic cation transporter 1 (OCT1) and the copper transporter 1 (CTR1), both involved in the uptake of cisplatin, have been found in some patients with CCA [34]. Low CTR1 tumor expression measured by immunohistochemistry has been associated with worse response to cisplatin in other solid tumors [27,28], but this question has not been investigated in CCA. An increased expression of glutathione S-transferase-pi (GSTP1–1), an enzyme involved in the metabolic inactivation of cisplatin, has been related to chemoresistance. Hence, specific GSTP1–1 inhibitors have been reported to reverse multidrug resistance phenotype in CCA cells [29]. Excision Repair Cross Complementation group 1 (ERCC1) is an essential component of Nucleotide Excision Repair (NER), one of the major mechanisms involved in repairing DNA-platinum adducts. High expression of ERCC1 in CCA tissues has been related to the lack of response to cisplatin [30]. Similar findings have been described for metallothioneins (MTs) [30], which regulate apoptosis and proliferation and protect DNA from the toxic effects of cisplatin [35]. High MT expression was associated with a lower effect of cisplatin in CCA using an in vitro histoculture drug response assay (HDRA) [30].

## 3. Response of CCA to Targeted Therapy

**Figure 2:**
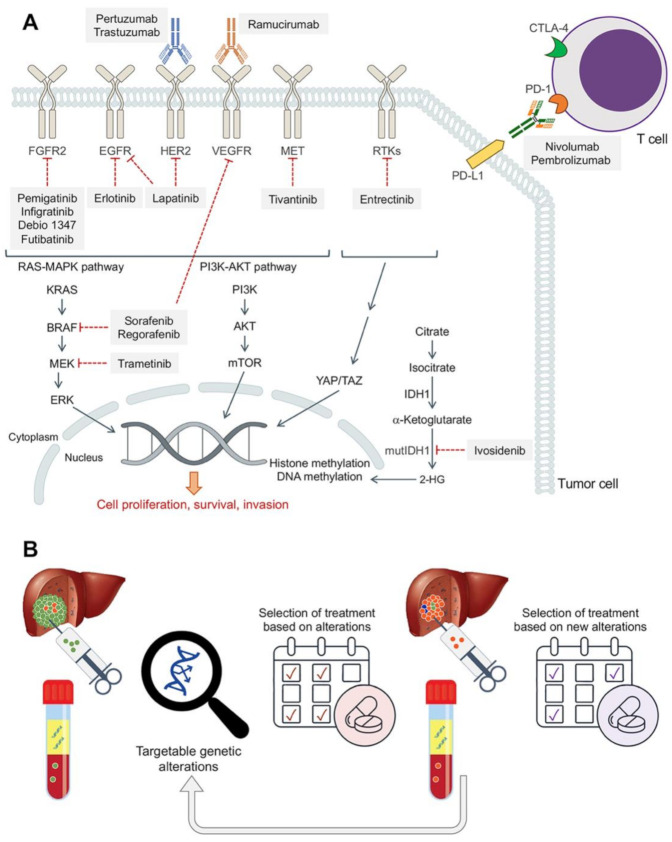
(**A**) Schematic representation of targeted therapies and immunotherapies that are being investigated in patients with CCA and are in more advanced studies. Resistance mechanisms have been described for some of them as has been discussed. (**B**) Schematic representation of the types of patient samples that can be used for molecular profiling to select the best targeted therapy for each patient at different times.

The use of drugs targeting vital molecular pathways of disease pathogenesis, including angiogenesis, provides good results in treating other types of cancer. Agents that selectively target several tyrosine kinases are preferred because tumor cells have been shown to activate different parallel transduction signals to survive (Figure 2A), and acting on numerous targets seems a more effective strategy.

In the last few years, the idea of identifying subgroups of cancer patients that might benefit from a personalized treatment has gained interest thanks to the availability of performing molecular profiling. This therapeutic approach can be especially advantageous in the case of CCA, since this is one of the tumors with the highest number of genetic alterations that are potentially druggable, which means they affect gene-encoding proteins that can be targeted directly or indirectly with approved or investigational drugs [36]. Several recent studies have described the current status of targeted therapies in clinical development in BTCs [2,37,38,39,40,41]. However, none of them have focused on information related to MPRs. Since resistance is a dynamic process, the obtention of subsequent tumor biopsies or performing analyses of circulating tumor DNA during disease progression can help to select an alternative drug or a combination of drugs that could permit to circumvent pharmacoresistance (Figure 2B). However, multiple and complex MPRs can occur in different subclones of a single tumor, making it challenging to reach a durable anticancer response.

### 3.1. Multikinase Inhibitors

The inhibition of cell surface and intracellular kinases involved in tumor growth and metastatic progression (Figure 2A) has proven successful in some cancers. Nevertheless, the beneficial effect of these drugs in CCA is low.

Sorafenib is an oral inhibitor of vascular endothelial growth factor receptors 2 and 3 (VEGFR-2/3), platelet-derived growth factor receptor β (PDGFR-β), and oncogenic kinases KIT, B-RAF, and C-RAF, that has shown a limited effect in advanced CCA as a single agent [42,43]. Poor efficacy of sorafenib has been partially attributed to low intracellular concentrations in tumor cells due to impaired OCT1-mediated uptake [44] and enhanced MRP3-mediated efflux [45,46] (Table 2).

Regorafenib inhibits angiogenic (VEGFR-1–3, TIE2) and stromal (PDGFR-β, fibroblast growth factor receptor 1 or FGFR1) factors that promote tumor vessel formation and suppresses several oncogenic kinases (KIT, RET, RAF) [56] and has shown promising results in advanced refractory CCA [47,57]. In patients included in that study, the elevated plasma VEGF-D levels were associated with shorter PFS, whereas IL-6 and glycoprotein 130 (GP130) levels were associated with shorter OS in CCA [47].

### 3.2. Fibroblast Growth Factor Receptor (FGFR) Inhibitors

FGFR family includes four members of transmembrane tyrosine kinase receptors (TKRs) involved in differentiation, proliferation, survival, migration, and angiogenesis [58]. The FGFR signaling pathway is frequently altered in all types of cancer. Thus, in iCCA, FGFR aberrations are common, particularly in FGFR2 (≈20%), with a predominance of rearrangements or fusions over amplifications [59]. Of note, some aberrant FGFR signaling has been associated with the resistance to anticancer agents. Since non-selective multi-tyrosine kinase inhibitors (TKIs), such as regorafenib, lenvatinib or pazopanib, have not been effective in CCA with FGFR aberrations [60], the next generation of drugs has included more selective TKIs, and some of them are being tested in CCA (Figure 2A).

Pemigatinib (INCB054828) is an oral inhibitor of FGFR1–3. Based on the results of the FIGHT-202 trial, which showed a 35.5% overall response rate (ORR) in 38 patients with FGFR2 fusions or rearrangements, three with a complete response and 35 with partial responses [61], the FDA approved in April 2020 this drug for chemotherapy-refractory CCA patients with FGFR2 rearrangements or fusions. The analysis of the genomic profiling and clinical results from patients prescreened and enrolled in the FIGHT-202 trial revealed interesting findings regarding drug resistance that need to be validated. Thus, co-occurring genomic alterations may be a mechanism of primary resistance to pemigatinib, and acquired mutations in the FGFR2 kinase domain (p.N549K/H, p.E565A, p.K659M, p.L617V, and p.K641R) were found in patients with an initial response but followed by disease progression [48].

Infigratinib (BGJ398) is an oral pan-inhibitor of FGFR (FGFR1–3 > FGFR4). A phase II study in selected FGFR-altered CCA patients that progressed after chemotherapy showed an ORR of 14.8% (18.8% FGFR2 fusions only) and a disease control rate (DCR) of 75.4% (83.3% FGFR2 fusions only) [62]. It has been described that acquired resistance to infigratinib can be caused by point-mutations in FGFR2, such as the gatekeeper mutation p.V564F, and by alterations in the PTEN/PI3K pathway [49].

Debio1347 is also an oral inhibitor of FGFR (FGFR1–3 > FGFR4). The results of the phase II FUZE trial in patients with advanced solid cancers (including CCAs) with FGFR genetic alterations are pending, but preliminary results are promising [63]. Preclinical studies showed that the presence of mutations p.N550K, p.L618V, and p.K660M in FGFR2 conferred resistance to Debio 1347 [50].

Futibatinib (TAS-120) is an oral irreversible pan-FGFR inhibitor (FGFR1–4). The phase I FOENIX-101 trial showed partial responses in FGFR2 fusion-positive iCCA patients who had shown refractoriness to standard chemotherapy [64]. Besides, promising results were obtained in iCCA patients with acquired resistance to other FGFR inhibitors [50]. Complementary functional studies in a panel of iCCA-derived cell lines expressing each of the described mutations confirmed that futibatinib retained efficacy against FGFR2 kinase domain mutations, except the p.V565F gatekeeper [50].

Erdafitinib (JNJ-42756493) is an oral pan-FGFR inhibitor (FGFR1–4) that showed a partial response in 3 of 11 CCA patients with an FGFR2-fusion or mutation [65], but there is not yet available information of mechanisms of resistance.

Derazantinib (ARQ 087) is a non-selective oral multi-TKI with a potent pan-FGFR (FGFR1–3 > FGFR4) activity that also targets PDGFR, KIT, RET, and SRC. A phase I/II trial with 29 patients with unresectable FGFR2 gene fusion-positive advanced iCCA showed a partial response of 20.7% and a DCR of 82.8% [66].

Although preliminary data with FGFR2 inhibitors have shown promising results in patients with FGFR2 fusions who failed the standard of care, more analyses are needed to understand better the MPRs, which will be required to develop more effective strategies in the future.

### 3.3. Epidermal Growth Factor Receptor (HER/EGFR/ERBB) Inhibitors

The HER family includes four members: epidermal growth factor receptor (EGFR, HER1, or ERBB1) and HER2–4, also known as ERBB2–4. The altered expression of HER2 and EGFR and the dysregulated signaling mediated by these receptors have been involved in iCCA pathogenesis [67]. Overexpression and amplification of HER2/3 are frequent in CCA, but more so in extrahepatic CCA (eCCA, ≈15%) than in iCCA (≈7%), which has led to the suggestion that they can be good targets for inhibition [68]. Several HER inhibitors, both TKIs and monoclonal antibodies, have been investigated as monotherapy or in combination with classical chemotherapeutic drugs; nevertheless, the results have been inconclusive [59,69].

The administration of erlotinib, an EGFR inhibitor, in combination with sorafenib as the first treatment in unselected patients with advanced biliary cancer, did not show satisfactory clinical activity [70]. Still, the addition of erlotinib to gemcitabine/oxaliplatin prolonged PFS versus chemotherapy alone in the subgroup of patients with advanced CCA [71]. To date, erlotinib has no value for biliary cancer patients in the clinic, but genomic profiling may allow the identification of patient subgroups for whom this drug could be beneficial [72]. Resistance to erlotinib in CCA has been associated with a change to a cancer stem cell (CSC)-like phenotype with the contribution of stromal cells [51].

The EGFR and HER2 inhibitor lapatinib showed no activity as a single agent in a reduced number of patients with biliary cancer. However, no overexpression of HER2 or mutations in these receptors were found in the analyzed tissue or blood [73].

Monoclonal antibodies pertuzumab and trastuzumab are both HER2 inhibitors. The combination of pertuzumab plus trastuzumab with chemotherapy is the first-line standard of care for HER2-positive metastatic breast cancer patients. It is showing better efficacy in patients with wild-type KRAS compared with KRAS-mutated disease [74]. This treatment is also being investigated in patients with advanced BTC with HER2 amplification or mutations that have previously received different lines of therapy. Preliminary data showed ORRs of 7.5% in patients with HER2 amplification and 33.3% in patients with HER2 mutations with good tolerability [75]. Four patients with HER2 mutation-bearing tumors unresponsive to previous treatments (one of them with advanced CCA) received trastuzumab plus lapatinib and experienced clinical benefit. The patient with CCA showed a PFS of 7.1 months, while a patient with colorectal adenocarcinoma that presented tumor shrinkage at first tumor assessment developed resistance, and the presence of the HER2 mutation p.L869R was detected in the metastatic tissue [52].

The HER2 downregulation at the plasma membrane level after treatment with trastuzumab/pertuzumab observed in other tumors has been associated with acquired resistance and cross-resistance [76]. However, this has not yet been investigated in CCA. The fact that some studies were not carried out in selected patients expressing the receptors requires new trials to confirm if targeting members of the HER family could be helpful in CCA pharmacotherapy. Future studies could also provide further insight into the mechanisms of resistance to these drugs.

### 3.4. Isocitrate Dehydrogenase (IDH) Inhibitors

IDHs are enzymes that catalyze the oxidative decarboxylation of isocitrate to α-ketoglutarate (αKG), a compound that promotes the activity of dioxygenases, which in turn epigenetically control gene expression. Mutations affecting IDH1 and IDH2 enzymes, located in the cytoplasm and the mitochondria, respectively, result in the accumulation of the oncometabolite D-2-hydroxyglutarate (2-HG), which leads to a global hypermethylation phenotype and hence impaired cellular differentiation [77]. Several studies have shown that ≈15–20% of iCCAs harbor *IDH1* mutations, especially in patients without liver fluke infection or hepatitis infection, while this only occurs in ≈1% of extrahepatic CCAs [78]. Moreover, *IDH1* mutations are more frequent than *IDH2* in iCCA.

Ivosidenib is a promising drug for patients harboring IDH1-mutated iCCA failing to respond to chemotherapy. This drug is a reversible oral inhibitor of mutant IDH1 that prevents the formation of 2-HG (Figure 2A). Phase I and III trials have shown that ivosidenib was well tolerated and improved PFS compared with placebo [79]. Several studies are underway using ivosidenib in combination with chemotherapy or immunotherapy [80].

Information on mechanisms of IDH1-mediated resistance in CCA is scarce. Switching between isoforms has been described as a mechanism of acquired resistance to mutant IDH inhibition in different tumors, including CCA. Thus, it has been reported the case of a patient with treatment-refractory IDH1 R132C-mutant iCCA who achieved a sustained partial response to ivosidenib, which was followed by disease progression associated with the acquisition of a new IDH2 R172V mutation [81]. Combined inhibition of IDH1 and IDH2 could be an option to prevent tumor cells from eluding the response to pharmacotherapy.

### 3.5. Neurotropic Tyrosine Kinase Receptor (NTKR) Inhibitors

*NTKR1–3* genes encode three tropomyosin receptor kinases (TRKs). Around 3.5% of patients with CCA present NTRK fusions, which constitutively activate these receptors and hence promote proliferation and survival.

Larotrectinib is a highly selective inhibitor of the three TRK forms approved by the FDA to treat solid tumors harboring an NTRK gene fusion based on the results of trials that showed an ORR of 75% and responses for one year; one of the two CCA patients included showed disease stabilization [82].

Entrectinib is also a potent inhibitor of the three TRK isoforms. This drug also targets ROS1 and ALK [83]. This drug was approved by the FDA to treat advanced NTRK gene fusion-positive solid tumors based on clinical trials demonstrating a durable ORR of 57% and responses for ten months [84]. In vitro and in vivo models of different types of cancer, which have not included CCA, have suggested that reactivation of RAF/MEK/ERK signaling accounts for resistance to entrectinib [54].

### 3.6. Other Targeted Therapies

Constitutive activation of the mitogen-activated protein kinase (MAPK) pathway is frequent in CCA. This pathway has been targeted with BRAF and MEK inhibitors. BRAF mutations occur in <5% of patients with biliary cancer [85]. The administration of the BRAF inhibitor dabrafenib and the MEK inhibitor trametinib in patients with the BRAF V600E mutation has shown promising results with a manageable safety profile in the subgroup of patients with biliary cancer [86]. In other tumors, it has been shown that reactivation of the MAPK pathway upstream of MEK is the major contributing factor for the resistance developed after treatment with this pharmacological combination [55].

Overexpression and amplification of MET have been found in CCA (<5%), which has been associated with poor prognosis [87]. The administration of MET inhibitors in CCA as monotherapy has shown low clinical efficacy. However, controversial results have been described in combination with other drugs. The oral selective MET inhibitor tivantinib (ARQ 197) has shown promising results when administered with gemcitabine in patients with metastatic tumors (including CCAs) [88]. In contrast, another oral inhibitor, merestinib, in combination with gemcitabine and cisplatin as first-line treatment, did not show an improvement in the survival of patients with advanced BTC [89].

## 4. Immunotherapy

Immunotherapy, which has emerged as a promising treatment strategy for many tumors, including CCA [90], is based on the use of drugs designed to interact with proteins involved in the crosstalk between cancer and immune cells [91]. Immune-checkpoint inhibitors (ICIs) target these cancer proteins aiming at preventing the binding with their partners located at the plasma membrane of immune system cells, such as cytotoxic T lymphocyte-associated antigen 4 (CTLA-4), programmed cell death 1 (PD-1), and its ligand, PD-L1 [91]. Although only two ICIs (nivolumab and pembrolizumab) have been approved for the treatment of biliary cancers [92], other immune-modulating therapies are currently being investigated.

Deficiencies in the DNA-mismatch repair (MMR) system, which can lead to microsatellite instability (MSI), are considered markers of enhanced response to ICIs, along with high tumor mutational burden, as they favor the formation of neoantigens and therefore their recognition by immune cells [93]. Although tumor mutational burden is high in biliary cancers, MMR deficiency or MSI are not frequent in these tumors [4].

### 4.1. Programmed Cell Death 1 (PD-1) Inhibitors

Nivolumab and pembrolizumab are monoclonal antibodies targeting PD-1 [94] (Figure 2A), a cell surface receptor present in immune cells, such as activated T-cells, B cells, and macrophages, that inhibits adaptive and innate immune responses and enables tumor cells to escape the attack by the immune system [95]. Both drugs have shown promising results in patients with BTC in different clinical trials with manageable toxicities. Thus, nivolumab alone showed an ORR of 11% and a DCR of 50% [96], whereas in the KEYNOTE-028 trial, including 28 PD-L1-positive patients, pembrolizumab showed an ORR and a DCR of 17% and 34%, respectively. These values were higher than those registered in a larger cohort of 104 unselected patients in the KEYNOTE-158 trial, with only 5.8% and 22.1%, respectively [97]. The ORR was improved (55.6%) if nivolumab was combined with chemotherapy [98].

In another study with PD-L1-positive gemcitabine/cisplatin-refractory BTC patients, a durable efficacy of pembrolizumab with an ORR of 9.8% was found [99]. Camrelizumab, another PD-1 inhibitor, combined with chemotherapy as first-line treatment of BTCs, showed promising results with 50% ORR and a median OS of 11.8 months [100]. The combination of pembrolizumab and ramucirumab, an antibody that targets VEGFR-2, also seemed to be beneficial as a second-line treatment for patients with advanced BTC, showing a reduction of tumor size of 37.5% and a DCR of 38.5% [101].

PD-L1 down-regulation has been associated with an unsatisfactory response to anti-PD-1 inhibitors when administered as monotherapy in solid tumors [90] (Table 3).

Using immunohistochemistry, several studies have shown that a low proportion of biliary tumors (5–9%) express PD-L1. In contrast, this ligand is expressed in the immune cells of the tumor microenvironment, mainly in tumor-associated macrophages (TAMs) [103,104,105]. A recent study in advanced refractory BTC patients treated with nivolumab demonstrated that lower levels of PD-L1 in tumors were associated with reduced PFS, but not with lower OS [96]. A similar result was observed in advanced BTC patients treated with camrelizumab plus GEMOX as a first-line treatment since the ORR was worse in patients with lower PD-L1 levels in tumors than in those with higher levels [100]. However, in two clinical trials, including patients with advanced biliary cancer, most of them refractory to other therapies, no correlation between PD-L1 levels and response to pembrolizumab monotherapy was found [97]). On the other hand, the combination of pembrolizumab and ramucirumab only improved the survival of patients with PD-L1-positive BTC, who reached a higher median OS (11.3 months) than that of those with PD-L1-negative tumors (6.1 months) [101].

In the KEYNOTE-158 study, a low tumor mutational burden correlated with a reduced response to pembrolizumab monotherapy [102]. In contrast, there was no association between the degree of response and this marker (determined in tissue or blood) in advanced BTC patients treated with camrelizumab plus GEMOX [100].

Several genetic alterations, which are common in CCA, have been related to changes in the tumor microenvironment that can impair anti-tumor immune responses [93]. For instance, alterations in Wnt/β-catenin signaling indirectly caused less infiltration of antigen-presenter dendritic cells. In comparison, the loss of PTEN was associated with less infiltration of T cells and resistance to a PD-1 blockade in melanoma [93].

Bintrafusp alfa is a bifunctional fusion protein composed of a human anti-PD-L1 antibody, which directs the drug to the tumor microenvironment, and the extracellular domain of the transforming growth factor β receptor (TGF-βRII), which “traps” the tumor-surrounding TGF-β [106], a cytokine involved in the regulation of cell cycle, apoptosis, self-tolerance and extracellular matrix composition [107]. Since TGF-β signaling is upregulated in CCA and involved in EMT [108], iCCA patients were treated with bintrafusp alfa in a phase I study, obtaining a 30% ORR. The clinical activity of this drug seems to be independent of MSI status and PD-L1 expression levels [109].

Changes in some immune cell populations, such as a lower proportion of baseline CD3+ T cells, and serum cytokines, such as higher levels of soluble FasL and interferon-γ (IFN-γ), were described as predictors of unsatisfactory outcome after ICI-based treatment of BTC patients [98].

### 4.2. Cytotoxic T Lymphocyte-Associated Antigen 4 (CTLA-4) Inhibitors

Ipilimumab is an antibody targeting CTLA-4, a cell surface protein that inhibits immune signaling by T cells [91]. It has been used in combination with nivolumab to treat CCA patients after chemotherapy failure, achieving a 23% ORR and a 44% DCR; all responders were iCCA patients that progressed after chemotherapy, and none had MSI [110].

CTLA-4 blockade enhances T-cell response against tumors through IFN-γ. Consequently, tumors lacking integrity of IFN-γ gene expression were more resistant to ipilimumab [111]. However, there is still no report on the effect of acting on this pathway in CCA.

Mechanisms to escape anti-tumor immune responses also include changes in immune-checkpoint proteins expression, the loss of Major Histocompatibility Complex (MHC), and the regulation of the tumor microenvironment towards an immunosuppressive milieu [90]. Notably, alterations in genes encoding components of the antigen processing and/or presentation system can lead to ICI resistance [93]. MHC-I downregulation has been found in BTC patients and was associated with lower lymphocyte infiltration [112]. However, altered HLA-I expression, found in some iCCA patients, helped the tumor scaping the immune system, which advises against the use of immunotherapy in iCCA patients with these characteristics [113].

Besides tumor defense mechanisms, the efficacy of ICI therapy relies on T-cell activity. For instance, CCA immunosuppressive microenvironment can prevent effector T-cell proliferation and activation [113]. Thus, the generation of insufficient anti-tumor T-cells, their inadequate function, or impaired memory formation can lead to poor response to immunotherapy [93].

## 5. Conclusions and Perspectives

In the last decade, many necessary steps have been taken to understand the cellular and molecular bases of the lack of response of CCA to pharmacological treatment. However, the picture remains little more than a draft, and only small advances have been reached in the search for effective drugs to treat this lethal form of cancer. The hope of developing new tools to improve the dismal outcome of these patients is now based on two lines of action: (i) the identification of specific druggable genetic alterations that can be pharmacologically manipulated with personalized, targeted anticancer agents, and (ii) the use of immunotherapy. The latter includes the development of small molecules and antibodies able to interact with proteins involved in the crosstalk between cancer and immune cells aiming at triggering the attack of the immune system against the tumor.

A better understanding of MPRs is essential to identify potential tumor Achilles’ heels that help to develop more effective drugs, as well as to select sequential treatments or combinations of drugs that hinder the survival of the most resistant cells. Significant efforts are being invested in creating methods to predict the response of individual tumors to a particular drug. In vitro assays using tumor cells isolated from surgically resected pieces and cultured as tumoroids or small fragments of tumors in culture are being used to obtain valuable information for personalized treatments, in some cases, with the help of artificial intelligence. Since tumor cells are not always available at different times of treatment, the analysis of circulating tumor cells, extracellular vesicles, and tumor DNA is of great interest to follow the evolution of acquired resistance to these therapies. In sum, future advances in the field of pharmacological treatment of CCA require a better understanding of different MRPs affecting classical chemotherapy, targeted therapy, and immunotherapy that could permit to exploit the existence of collateral sensitivity and the use of potential synergistic effects in the design of personalized treatments.

## Figures and Tables

**Table 1:** MPRs to classical chemotherapy in CCA.

Class	Drug	Mechanisms of Resistance	Effect	Preclinical Model	Ref.
Antimetabolites	Gemcitabine	Reduced ENT1 levels/impaired plasma membrane targeting	Lower PFS	-	[16,17]
		High MRP1 expression	Reduced effect *	Resistant cell lines	[18]
		Decreased DCK activity	Reduced effect *	Resistant cell lines	[18]
		High P53R2 expression	Reduced effect *	Cell lines	[19]
		KPNA2 overexpression	Lower OS	-	[20]
		Bcl-2 upregulation/Bax downregulation	Reduced effect *	Resistant cell lines	[18]
		Higher MMP-9/uPA activity	Reduced effect *	Resistant cell lines	[18]
		Oct4 upregulation	Reduced effect *	Cell lines	[21]
		High HMGA1 expression	Reduced PFS	-	[22]
		High IL-6/TGF-β1 expression	Reduced effect *	Cell lines	[23]
	5-FU	Low OPRT expression	Reduced effect *	Isolated tumor cells	[24]
		High TS expression	Reduced effect *	Cell lines	[25]
		NK4 downregulation	Reduced effect *	Cell lines	[26]
Alkylating agents	Cisplatin	Reduced CTR1 expression	Lower OS ^†^	-	[27,28]
		Increased GSTP1–1 expression	Reduced effect *	Cell line	[29]
		High ERCC1 expression	Reduced effect *	Isolated tumor cells	[30]
		High MT expression	Reduced effect *	Isolated tumor cells	[30]

5-FU, 5-Fluorouracil; DCK, deoxycytidine kinase; ENT-1, equilibrative nucleoside transporter 1; ERCC1, excision repair cross complementation group 1; GSTP1–1, glutathione S-transferase-pi; HMGA1, high mobility group A1; IL-6, interleukin 6; KPNA2, Karyopherin-α2; MMP-9, matrix metalloproteinase-9; MRP1, multidrug resistance-associated protein 1; MT, metallothionein; Oct4, octamer-binding transcription factor 4; OPRT, orotate phosphoribosyl transferase; OS, overall survival; P53R2, p53-inducible ribonucleotide reductase; PFS, progression-free survival; TGF-β1, transforming growth factor β1; TS, thymidylate synthase; uPA, urokinase plasminogen activator. *, predicted from preclinical studies. ^†^, predicted from clinical studies in other tumors.

**Table 2:** MPRs to targeted therapy in CCA.

Inhibit	Drug	Target/s	Mechanisms of Resistance	Effect	Ref.
Kinases	Sorafenib	VEGFR-2/3, PDGFR-β, KIT, B-RAF, C-RAF	Reduced uptake, Increased efflux	Low efficacy *	[44,45]
	Regorafenib	VEGFR-1–3, TIE2, PDGFR-β, FGFR1, KIT, RET, RAF	High VEGF High IL-6, GP130	Reduced PFS Reduced OS	[47]
FGFR	Pemigatinib	FGFR1–3	Mutations in FGFR2	Progression	[48]
	Infigratinib	FGFR1–3 > FGFR4	FGFR2 mutations and altered PTEN/PI3K pathway	Progression	[49]
	Debio 1347	FGFR1–3 > FGFR4	Mutations in FGFR2	Progression *	[50]
	Futibatinib	FGFR1–4	Not described	Unknown	
	Erdafitinib	FGFR1–4	Not described	Unknown	
	Derazantinib	FGFR1–3 > FGFR4, PDGFR, KIT, RET, SRC	Not described	Unknown	
HER	Erlotinib	EGFR	Induced CSC-like phenotype	Lower response *	[51]
	Lapatinib	EGFR, HER2	Not described	Unknown	
	Pertuzumab	HER2	Not described	Unknown	
	Trastuzumab	HER2	Mutations in HER2	Progression	[52]
IDH	Ivosidenib	Mutant IDH1	RTK pathway mutations, 2-HG-restoring mutations	Progression ^†^	[53]
NTKR	Larotrectinib	TRK1–3	Not described	Unknown	
	Entrectinib	TRK1–3, ROS1, ALK	Reactivation of RAF→ MEK→ ERK signaling	Progression ^†^	[54]
BRAF	Dabrafenib	BRAF ^V600E^ mutation	Reactivation of MAPK pathway upstream of MEK	Progression ^†^	[55]
MEK	Trametinib	MEK			
MET	Tivantinib	MET	Not described	Unknown	

2-HG, D-2-hydroxyglutarate; CSC, cancer stem cell; EGFR (or HER), epidermal growth factor receptor; FGFR, fibroblast growth factor receptor; GP130, glycoprotein 130; IDH, isocitrate dehydrogenase; IL-6, interleukin 6; MAPK, mitogen-activated protein kinase; NTKR, neurotropic tyrosine kinase receptor; OS, overall survival; PDGFR, platelet-derived growth factor receptor; PFS, progression-free survival; PTEN/PI3K, phosphatase and tensin homolog/phosphoinositide 3-kinase; RTK, receptor tyrosine kinase; TRK, tropomyosin receptor kinase; VEGFR, vascular endothelial growth factor receptor. *, predicted from preclinical studies; ^†^, predicted from clinical studies in other tumors.

**Table 3:** MPRs to immunotherapy in CCA.

PD-1 Inhibitor	Mechanisms of Resistance	Effect	Ref.
Nivolumab	PD-L1 down-regulation	Reduced PFS	[96]
Camrelizumab		Worse ORR	[100]
Pembrolizumab (+ ramucirumab)		Reduced OS	[101]
Pembrolizumab	Low tumor mutational burden	Worse ORR	[102]
Nivolumab (+ GEM/CIS)	Changes in immune cells	Worse outcome	[98]

GEM/CIS, gemcitabine and cisplatin; ORR, overall response rate; OS, overall survival; PD-L1, programmed cell death ligand-1; PFS, progression-free survival.

## Abbreviations

BTC: biliary tract cancer; CCA, cholangiocarcinoma; FGFR, fibroblast growth factor receptor; iCCA, intrahepatic CCA; IDH, isocitrate dehydrogenase; MPR, mechanism of pharmacoresistance; ORR, overall response rate; OS, overall survival; PDGFR, platelet-derived growth factor receptor; PFS, progression-free survival; RTK, receptor tyrosine kinase; TKI, tyrosine kinase inhibitor; TRK, tropomyosin receptor kinase; VEGF, vascular endothelial growth factor; VEGFR, vascular endothelial growth factor receptor.
